# In Silico Analysis and Validation of A Disintegrin and Metalloprotease (ADAM) 17 Gene Missense Variants: Structural Bioinformatics Study

**DOI:** 10.2196/72133

**Published:** 2025-08-25

**Authors:** Abdelilah Mechnine, Asmae Saih, Lahcen Wakrim, Ahmed Aarab

**Affiliations:** 1 Biotechnology and Biomolecule Engineering Research Team, Faculty of Sciences and Techniques of Tangier, Abdelmalek Essaâdi University, Ancienne Route de l'Aéroport, Km 10, Ziaten. BP:416, Tangier, 90030, Morocco; 2 Laboratory of Biology and Health, URAC 34, Faculty of Sciences Ben M'Sik, University of Hassan II Casablanca, Casablanca, Morocco; 3 Virology Unit, Immunovirology Laboratory, Institut Pasteur du Maroc, Casablanca, Morocco

**Keywords:** bioinformatics, in silico, COVID-19, SARS-CoV-2, molecular modeling

## Abstract

**Background:**

The protein A disintegrin and metalloprotease (ADAM) domain containing 17, also called tumor necrosis factor alpha–converting enzyme, is mainly responsible for cleaving a specific sequence Pro-Leu-Ala-Gln-Ala-/-Val-Arg-Ser-Ser-Ser in the membrane-bound precursor of tumor necrosis factor alpha. This cleavage process has significant implications for inflammatory and immune responses, and recent research indicates that genetic variants of ADAM17 may influence susceptibility to and severity of SARS-CoV-2 infection.

**Objective:**

The aim of the study is to identify the most deleterious missense variants of ADAM17 that impact protein stability, structure, and function and to assess specific variants potentially involved in SARS-CoV-2 infection.

**Methods:**

A bioinformatics approach was used on 12,042 single-nucleotide polymorphisms using tools including SIFT (Sorting Intolerant From Tolerant), PolyPhen2.0, PROVEAN (Protein Variation Effect Analyzer), PANTHER (Protein Analysis Through Evolutionary Relationships), SNP&GO (Single Nucleotide Polymorphisms and Gene Ontology), PhD-SNP (Predictor of Human Deleterious Single Nucleotide Polymorphisms), Mutation Assessor, SNAP2 (Screening for Non-Acceptable Polymorphisms 2), MUpro, I-Mutant, iStable, InterPro, Sparks-x, PROCHECK (Programs to Check the Stereochemical Quality of Protein Structures), PyMol, Project HOPE (Have (y)Our Protein Explained), ConSurf, and SWISS-MODEL. Missense variants of ADAM17 were collected from the Ensembl database for analysis.

**Results:**

In total, 7 nonsynonymous single-nucleotide polymorphisms (P556L, G550D, V483A, G479E, G349E, T339P, and D232E) were identified as high-risk pathogenic by all prediction tools, and these variants were found to potentially have deleterious effects on the stability, structure, and function of the ADAM17 protein, potentially destroying the entire cleavage process. Additionally, 4 missense variants (Q658H, D657G, D654N, and F652L) in positions related to SARS-CoV-2 infection exhibited high conservation scores and were predicted to be deleterious, suggesting that they play an important role in SARS-CoV-2 infection.

**Conclusions:**

Specific missense variants of ADAM17 are predicted to be highly pathogenic, potentially affecting protein stability and function and contributing to SARS-CoV-2 pathogenesis. These findings provide a basis for understanding their clinical relevance, aiding in early diagnosis, risk assessment, and therapeutic development.

## Introduction

The ADAM family, which stands for A disintegrin and metalloprotease*,* is made up of both single-passage transmembrane proteins and secreted metalloendopeptidases. These enzymes share a distinct domain structure, which includes a prodomain, metalloprotease domain, disintegrin domain, cysteine-rich region, epidermal growth factor–like domain, a transmembrane segment, and a C-terminal cytoplasmic tail [1,2].

However, some human ADAM proteins lack a functional protease domain, meaning that many of ADAMs’ roles are centered on protein-protein interactions rather than protease activity. ADAM proteins belong to the EC 3.4.24.46 enzyme classification and are part of the MEROPS M12B peptidase family. For instance, active ADAM proteases are often referred to as sheddases because they cleave or remove extracellular parts of transmembrane proteins, such as ADAM10, and are able to cleave part of the human epidermal growth factor receptor 2, which then activates the receptor. ADAM genes are present in choanoflagellates, animals, fungi, and certain green algae, while these proteins are not present in most green algae and all land plants because they probably lost it. ADAM proteins have been historically referred to by names like adamalysin or MDC (metalloproteinase type, disintegrin type, cysteine-rich) family [3-6].

ADAM17 is a polypeptide of 824 amino acids, 93,021 Da, and it is located on chromosome 2p25. ADAM17 is hugely expressed in a lot of tissues, such as the brain, kidney, heart, and voluntary muscle, and its expression changes during embryonic development and adult life. ADAM17 is a multidomain protein composed of several conserved domains, starting with an N-terminal signal peptide spanning amino acids (aa 1‐17), followed by a prodomain (aa 18‐216), in which there is a cysteine switch-like region PKVCGY^186^ (aa 181‐188), a metalloenzyme or catalytic domain (aa 217‐474) with a Zn-binding domain region (aa 405‐417), a disintegrin cysteine-rich domain (aa 480‐559), an epidermal growth factor–like region (aa 571‐602), followed by a cysteine-rich domain (aa 603‐671), and a transmembrane domain (aa 672‐694), end by a cytoplasmic tail (aa 695‐824). Tyr^702^, Thr^735^, and Ser^819^ have been shown as cytoplasmic phosphorylation sites, and Ser^791^ has been shown as a cytoplasmic dephosphorylation site. ADAM17 has little or no sequence similarities with other ADAMs, its closest relative is ADAM10; however, their protein sequence homology is a smaller amount than 30% consistent with the National Center for Biotechnology Information Basic Local Alignment Search Tool [7,8].

The purpose of ADAM17 is to treat tumor necrosis factor alpha (TNF-α) both inside the trans-Golgi network’s internal membranes and on the cell’s surface. The cleavage and release of a soluble ectodomain from membrane-bound proproteins (such as pro-TNF-α) involve this process, which is also known as “excretion” and is recognized to have physiological significance. The first “sheddase” to be discovered, ADAM17, is also thought to be involved in the release of a wide range of membrane-anchored cytokines, cell adhesion molecules, receptors, ligands, and enzymes [9,10].

The 26-kDa type II transmembrane propolypeptide that the TNF-α gene encodes inserts into the cell membrane during maturation, according to the gene’s cloning. Pro-TNF-α is physiologically active on the cell surface and can trigger immunological responses by means of juxtacrine intercellular communication. The Ala76-Val77 amide bond of pro-TNF-α, however, is susceptible to proteolytic breakage, which liberates the molecule’s soluble 17-kDa extracellular domain (ectodomain). The cytokine known as TNF-α, which is of vital importance in paracrine signaling, is the soluble ectodomain. ADAM17 catalyzes the proteolytic release of soluble TNF-α [11].

ADAM17 has recently been identified as a key modulator of radiation therapy resistance. Radiation treatment may induce furin-mediated cleavage of the inactive form of ADAM17, converting it into its active form in a dose-dependent manner. This results in increased ADAM17 activity both in vitro and in vivo. In nonsmall cell lung cancer, radiation therapy has also been demonstrated to activate ADAM17, which leads to the excretion of several survival factors, the activation of the growth factor pathway, and the development of radiation resistance [12].

In addition, ADAM17 might be a key player in the Notch signaling pathway when the intracellular Notch domain (from the Notch1 receptor) is released proteolytically following ligand interaction. By controlling the mammary gland’s excretion of the epidermal growth factor receptor, amphiregulin ligand, ADAM17 also controls the mitogen-activated protein kinase signaling pathway. Additionally, ADAM17 contributes to the excretion of the cell adhesion protein, L-selectin. To investigate the structural and functional effects of the chosen missense variations of the ADAM17 protein, we used a variety of bioinformatic techniques in the current methodology [13,14].

The primary cellular receptor used by SARS-CoV-2 to infect cells is the enzyme angiotensin-converting enzyme 2 (ACE2). This receptor is recognized by the S protein of SARS-CoV-2, which facilitates the key process of viral entry into a target cell. ADAM17 directly interacts with ACE2, leading to the shedding of ACE2 into the extracellular space, while transmembrane protease, serine 2 (TMPRSS2) not only cleaves ACE2 but also cleaves the SARS-CoV-2 S protein, facilitating membrane fusion and cellular uptake of the virus.

Both ADAM17 and TMPRSS2 act on ACE2, although these proteases can have opposite effects on the loss of ACE2. When the respective proteolytic activities of ADAM17 and TMPRSS2 result in increased shedding of ACE2, this situation may act as a natural barrier to infection. This could be due to the interaction of soluble ACE2 with the virus, preventing it from binding to susceptible tissues [15-21].

Alongside our work on ADAM17 variants and SARS-CoV-2 infection, Cho et al [22] explored in detail the immunogenicity of COVID-19 vaccines in different patients and highlighted immune response variation in terms of COVID-19 host factors. Additionally, Abbas et al [23] have used machine learning to profile RNA 5-methylcytosine modifications, a computational approach that is similarly conceptually related to how we have applied predictive methods in our analysis of ADAM17 variants [23].

This study highlights the potential of bioinformatics-driven variant analysis in exploring high-risk ADAM17 mutations, shedding light on their possible role in SARS-CoV-2 infection and advancing our understanding of ADAM17’s impact on immune and inflammatory processes.

## Methods

### Overview

We collected single-nucleotide polymorphisms (SNPs) of the ADAM17 gene data from the Ensembl database [24]. Only missense variants were extracted from the total SNPs for the first study, and only 7 missense variants were selected and tested for further bioinformatic approaches. For the second study, only variants related to SARS-CoV-2, located between positions 652 and 658, were extracted. In total, 4 missense variants were selected and tested using bioinformatics approaches. The amino acid sequence in FASTA (FAST-All) format was retrieved from the UniProt database [25-27].

### Ethical Considerations

This study involved only in silico analyses based on publicly available genomic data retrieved from the Ensembl genome database [24]. The data used are fully anonymized and do not contain any personally identifiable information or involve human or animal subjects. Thus, no ethics approval was required.

### Prediction of Deleterious Nonsynonymous SNPs Using SIFT, PolyPhen, PROVEAN, SNAP2, Mutation Assessor, PANTHER, SNP&GO, and PhD-SNP

We used 5 bioinformatic servers for the first study, namely, SIFT (Sorting Intolerant From Tolerant), PolyPhen, PROVEAN (Protein Variation Effect Analyzer), SNAP2 (Screening for Non-Acceptable Polymorphisms 2), and Mutation Assessor. SIFT (version 6.0), a web-based server, was used to predict the impact of a substitution on protein function. A SIFT score >0.05 indicates a tolerated or neutral mutation, while a score <0.05 indicates a deleterious or damaging mutation. PolyPhen-2 (version 2.2) is a web server that predicts the impact of mutations on protein structure and function. It was used to classify mutations into probably damaging, possibly damaging, and neutral. PROVEAN (version 1.1) is a web-based tool that analyzes the functional impact of protein mutations. When the score >2.5, the mutation is considered as neutral and has no effect on the protein. When the score is <2.5, the mutation is considered as deleterious and consequently has a deleterious effect on the protein. SNAP2 is a web-based server that is used to predict the functional effect of a mutation. Based on a neural network method, SNAP2 predicts the changes due to a nonsynonymous single-nucleotide polymorphism (nsSNP) on the secondary structure and compares the solvent accessibility of the native and mutated protein to distinguish them into effect (+100, strongly predicted) or neutral (−100, strongly predicted). Mutation Assessor is a web-based tool that is used to predict the functional effect of a mutation on a protein based on an evolutionary conservation approach. We used 5 bioinformatic servers for the second study, namely, SIFT, PolyPhen, PANTHER (Protein Analysis Through Evolutionary Relationships), SNP&GO (Single Nucleotide Polymorphisms and Gene Ontology), and PhD-SNP (Predictor of Human Deleterious Single Nucleotide Polymorphisms). PANTHER is a web-based tool for predicting nonsynonymous genetic variants that may play a causal role in human disease. PANTHER includes the Position-Specific Evolutionary Preservation tool, which predicts deleterious or pathogenic variants based on evolutionary conservation across homologous proteins from various organisms. The reference protein sequence of humans as well as sequences from 100 other species is used for these predictions. SNP&GO is a web-based tool using support vector machine (SVM) methods to predict whether a mutation is disease-related based on the protein sequence. The protein sequence is formatted in FASTA, and results are categorized as either neutral or disease-related, with a reliability index greater than 5 indicating a disease-causing mutation. PhD-SNP is also based on SVM and predicts whether a point mutation is a neutral polymorphism or associated with genetic disorders. It uses unique information derived from protein sequence, phylogenetic relationships, and the protein’s encoded function to determine whether the variant is disease-associated. This part was inspired by a study by Saih et al [28], who used SIFT, PolyPhen, and PROVEAN consecutively [29-36].

### Prediction of Mutation Effect on Stability and Structure of ADAM17 Protein Using I-Mutant, MUpro, and iStable

I-Mutant is a predictor of the effect of a single mutation on protein stability using protein sequences or structures and is an SVM tool based on predicting automatically the stability changes of a protein upon single-point mutations. A delta G>0 indicates a decrease in protein stability, while delta G<0 suggests increased stability. MUpro is a web-based tool used to predict the effect of mutations on the stability (increase or decrease) of a protein. The score >0 means that the mutation results in an increase in the stability of the protein, while a score <0 means that the mutation decreases the stability of the protein. iStable (Integrated predictor for protein stability change upon single mutation) analyzes protein stability using sequence information and predictions from different predictors. In this sequential analysis, 3 predictors are used: I-Mutant2.0, MUpro, and iStable [37-39].

### Conservation and Conserved Domain Analysis Using ConSurf and InterPro

ConSurf is a web server that is used for estimating the evolutionary conservation of amino or nucleic acid positions in a protein or DNA or RNA molecule based on the phylogenetic relations between homologous sequences and also for identifying functional regions. A conservation score ranging from 1 to 3 is considered variable, 5 to 6 is intermediate, and 7 to 9 indicates high conservation. InterPro is a web-based server that is used to identify the location of nsSNPs on conserved domains. InterPro recognizes protein motifs and domains, enabling functional characterization of the protein using its database of protein families, domains, and functional site [40-43].

### ADAM17 Modeling Using SWISS-MODEL Server and Sparks-X

SWISS-MODEL is a web server dedicated to protein structure homology modeling at different levels of complexity. 3D protein structures provide valuable insights into their molecular function and inform a broad spectrum of applications in life science research. Modeling of protein structures usually requires extensive expertise in structural biology and the use of highly specialized computer programs for each of the individual steps of the modeling process, and templates selected based on sequence identity and Global Model Quality Estimation score. Sparks-x is a fold-recognition method used to generate 3D protein structures. This tool improves structure prediction through enhanced alignment scoring and the use of SPINE-X, which boosts predictions of secondary structure, backbone torsion angles, and solvent-accessible surfaces [44,45].

### Validation of ADAM17 Models Using PROCHECK

PROCHECK (Programs to Check the Stereochemical Quality of Protein Structures) assesses the stereochemical quality of protein structures. It produces PostScript plots analyzing the global and residue-level geometry. PROCHECK-NMR is used to check the quality of structures resolved by nuclear magnetic resonance [46,47].

### Prediction of Mutation Effect on Protein Structure Using Project HOPE Server

HOPE (Have (y)Our Protein Explained) server is based on the automatic analysis of mutants, which can provide more clarification of the structural and functional effects on it. HOPE is an application that analyzes mutations automatically and explains the molecular source (origin) of a disease caused by it [48].

### Visualization of ADAM17 Native and Mutants Using PyMol

PyMol (version 2.5) is a molecular visualization program used to generate high-quality 3D images of proteins, as well as to edit molecular structures, perform ray tracing, and create molecular animations. PyMol (version 1.2r3pre; Schrödinger, LLC) is written in Python, one of the most popular programming languages, and can be easily extended through Python-based plugins.

All computational analyses were performed using default parameters except where otherwise noted. Tools were accessed from March to November 2024.

## Results

### Overview

In the first study, a total of 12,042 SNPs were collected from the Ensembl database. PROVEAN, PolyPhen-2, Mutation Assessor, SNAP2, and SIFT programs were used to predict the functional effects of mutations on ADAM17, while MUpro and I-Mutant tools were used to predict the mutation effects on protein stability. Additionally, SWISS-MODEL, ConSurf, and HOPE project were used to evaluate the mutation effects on protein function, structure, and protein-protein interactions. Ten various bioinformatics programs and tools are used to predict the mutation effects during this analysis, as relying on a single program or server is insufficient for accurately assessing mutation impact on proteins.

Among all collected SNPs, only those variants related to SARS-CoV-2 (positions 652 to 658) were extracted. Four missense variants were selected and analyzed using bioinformatics approaches.

### Prediction of Deleterious nsSNPs Using SIFT, PolyPhen, PROVEAN, SNAP2, Mutation Assessor, PANTHER, SNP&GO, and PhD-SNP

Of the initial 12,042 SNPs analyzed to predict deleterious effects on the ADAM17 protein, all were first submitted to SIFT; according to SIFT, 88 of these mutations were predicted to be deleterious (index score from 0 to 0.02). These 88 SNPs were subsequently analyzed with PROVEAN, and the results of PROVEAN showed that 60 SNPs were predicted deleterious. Similarly, when analyzed with PolyPhen, 48 of the SNPs were found to be probably damaging, with scores >0.9.

Next, the same 88 SNPs were analyzed using SNAP2, which predicted that 75 SNPs would have functional impacts on the ADAM17 protein with a score more than 1. Finally, Mutation Assessor identified 66 SNPs with a medium functional impact. In summary, from all 12,042 SNPs, only 7 mutations, namely, P556L, G550D, V483A, G479E, G349E, T339P, and D232E, were predicted to have a high functional impact on the ADAM17 protein by all computational tools (Tables1  2).

**Table 1.** Prediction of deleterious nonsynonymous single-nucleotide polymorphisms of the ADAM17^a^ gene using SIFT^b^ and PolyPhen.

Variant ID	Amino acid mutation	SIFT score	SIFT class	PolyPhen score	PolyPhen class
rs1394373815	P 556 L	0	Deleterious	0.997	Probably damaging
rs542316178	G 550 D	0	Deleterious	0.987	Probably damaging
rs777478676	V 483 A	0	Deleterious	0.987	Probably damaging
rs951262662	G 479 E	0	Deleterious	0.996	Probably damaging
rs1192348585	G 349 E	0.01	Deleterious	1	Probably damaging
rs1157021454	T 339 P	0	Deleterious	1	Probably damaging
rs768704961	D 232 E	0	Deleterious	1	Probably damaging

a ADAM: A disintegrin and metalloprotease.

b SIFT: Sorting Intolerant From Tolerant.

**Table 2.** Prediction of deleterious nonsynonymous single-nucleotide polymorphisms of ADAM17^a^ gene using PROVEAN^b^, Mutation Assessor, and SNAP2^c^.

Variant ID	Amino acid mutation	PROVEAN		Mutation Assessor		SNAP2	
Score	Prediction	Score	Prediction	Score	Prediction
rs1394373815	P 556 L	−8.978	Deleterious	4.095	High	37	Effect
rs542316178	G 550 D	−6.068	Deleterious	4.83	High	76	Effect
rs777478676	V 483 A	−3.496	Deleterious	4.215	High	43	Effect
rs951262662	G 479 E	−7.119	Deleterious	4.78	High	89	Effect
rs1192348585	G 349 E	−7.269	Deleterious	3.83	High	83	Effect
rs1157021454	T 339 P	−5.606	Deleterious	3.735	High	69	Effect
rs768704961	D 232 E	−3.795	Deleterious	3.79	High	84	Effect

a ADAM: A disintegrin and metalloprotease.

b PROVEAN: Protein Variation Effect Analyzer.

c SNAP2: Screening for Non-Acceptable Polymorphisms 2.

For the second study, 4 SARS-CoV-2–related nsSNPs (Q658H, D657G, D654N, and F652L) were submitted to SIFT. According to SIFT, mutations Q658H, D657G, and D654N were predicted to be deleterious (with index scores between 0 and 0.01), and F652L to be tolerated.

PolyPhen classified all 4 mutations as benign, while PANTHER identified D657G and D654N as likely damaging (score>0.57), and Q658H and F652L as possibly damaging (score~0.5).

The 4 nsSNPs were submitted to the SNP&GO program, which indicated that these SNPs would not have effects related to human diseases with scores above 0. The same nsSNPs were also analyzed using PhD-SNP, where the results indicated that the mutation D657G might have a pathogenic impact with a score of 3, while the other 3 mutations (Q658H, D654N, and F652L) were predicted to have neutral impacts (Tables3  4).

**Table 3.** Prediction of the deleterious effects of nonsynonymous single-nucleotide polymorphisms related to SARS-CoV-2 using SIFT^a^ and PolyPhen.

Variant ID	Amino acid mutation	SIFT score	SIFT class	PolyPhen score	PolyPhen class
rs765452935	Q658H	0.01	Deleterious	0.003	Benign
rs144657795	D657G	0	Deleterious	0.097	Benign
rs758594009	D654N	0	Deleterious	0.063	Benign
rs780262610	F652L	0.81	Tolerated	0.015	Benign

a SIFT: Sorting Intolerant From Tolerant.

**Table 4.** Prediction of the deleterious effects of nonsynonymous single-nucleotide polymorphisms related to SARS-CoV-2 using PANTHER^a^, SNP&GO^b^, and PhD-SNP^c^.

Variant ID	Amino acid mutation	PANTHER			SNP&GO		PhD-SNP	
		PSEP^d^	Prediction	*P*del	Reliability index	Prediction	Reliability index	Prediction
rs765452935	Q658H	220	Possibly damaging	.50	6	Neutral	3	Neutral
rs144657795	D657G	455	Probably damaging	.57	2	Neutral	3	Disease
rs758594009	D654N	1036	Probably damaging	.85	1	Neutral	5	Neutral
rs780262610	F652L	220	Possibly damaging	.50	8	Neutral	6	Neutral

a PANTHER: Protein Analysis Through Evolutionary Relationships.

b SNP&GO: Single Nucleotide Polymorphisms and Gene Ontology.

c PhD-SNP: Predictor of Human Deleterious Single Nucleotide Polymorphisms.

d PSEP: Position-Specific Evolutionary Preservation.

### Prediction of Mutation Effects on the Protein Energy and Stability Using I-Mutant, MUpro, and iStable Servers

MUpro results showed that 6 of the 7 selected mutations (G550D, V483A, G479E, G349E, T339P, and D232E) were predicted to decrease the stability of the ADAM17 protein, while the mutation P556L was predicted to increase the stability of the ADAM17 protein. Then, the 7 selected mutations were submitted to I-Mutant. Results of I-Mutant showed that all the 7 mutations (P556L, G550D, V483A, G479E, G349E, T339P, and D232E) were predicted to decrease the stability of ADAM17 protein (Table 5).

**Table 5.** Prediction of ADAM17^a^ stability using MUpro and I-Mutant tools.

Mutation	MUpro		I-Mutant	
Delta G	Prediction	Delta G	Prediction
P 556 L	0.20231915	Increase	−0.51	Decrease
G 550 D	−0.35202875	Decrease	−0.76	Decrease
V 483 A	−2.5029256	Decrease	−1.4	Decrease
G 479 E	−0.20347724	Decrease	−0.8	Decrease
G 349 E	−0.096611232	Decrease	−0.48	Decrease
T 339 P	−1.4271878	Decrease	−0.63	Decrease
D 232 E	−1.1774927	Decrease	−0.58	Decrease

a ADAM: A disintegrin and metalloprotease.

For the second study, iStable was used to predict the stability of these 4 mutations on the ADAM17 protein. The iStable results indicated that these 4 residues (Q658H, D657G, D654N, and F652L) were predicted to decrease the protein’s stability (Table 6).

**Table 6.** Stability analysis of ADAM17^a^ mutations related to SARS-CoV-2 using iStable.

Mutation	Confidence score	Prediction
Q658H	0.671109	Decrease
D657G	0.846768	Decrease
D654N	0.799807	Decrease
F652L	0.808582	Decrease

a ADAM: A disintegrin and metalloprotease.

### Prediction of Phylogenetic Conservation Using ConSurf and Study of Conserved Domains Using InterPro

The ConSurf analysis showed that all 7 substitutions are extremely conserved with a conservation score of 9. Six of these mutations (P556L, G550D, V483A, G479E, T339P, and D232E) were predicted to be exposed and functional, while the mutation G349E was predicted to be buried and structural. In the second study, ConSurf results showed 3 substitutions (Q658H, D657G, and D654N) to be highly conserved with a score of 8 and were predicted to be exposed and functional, while substitution (F652L) was very conserved with a score of 7 and predicted to be buried. The full visualization of the ConSurf-based phylogenetic conservation analysis of ADAM17 is provided in Multimedia Appendix 1.

**Figure 1.**
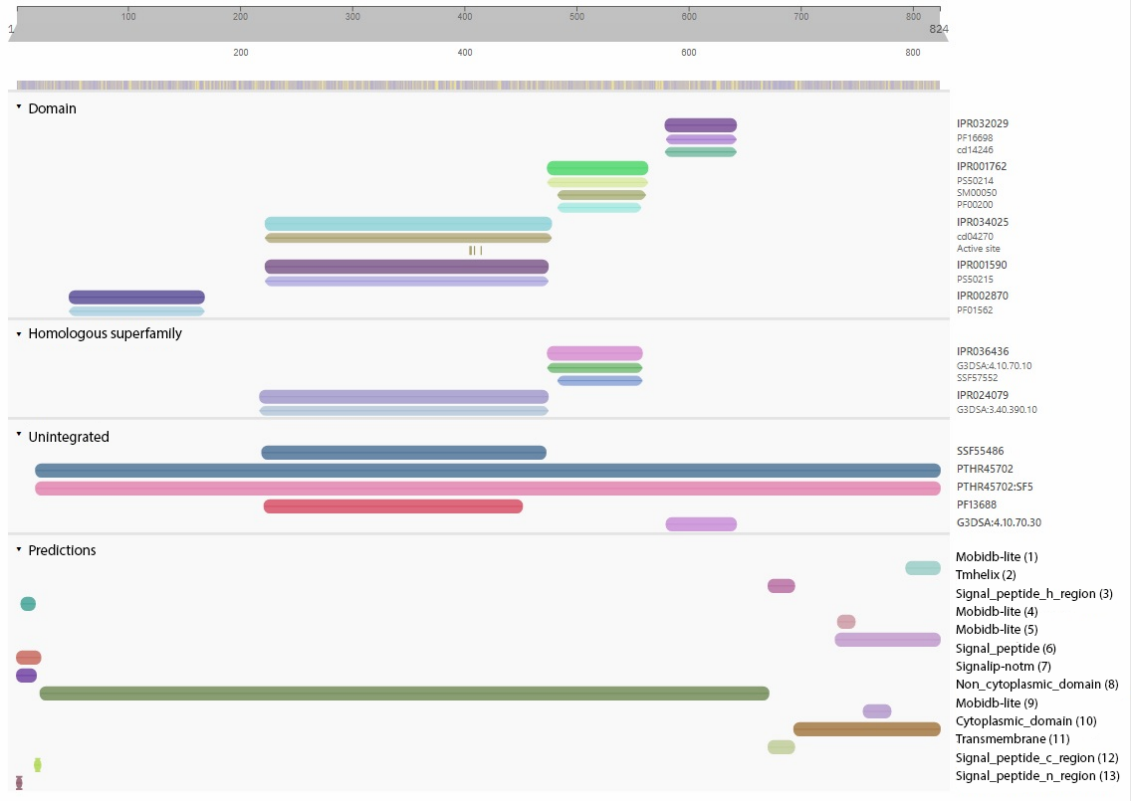
Identification of the ADAM17 protein domain using the InterPro server. ADAM: A disintegrin and metalloprotease.

For the second study, the InterPro domains identified include IPR032029, which indicates the ADAM17 proximal membrane domain (580-642), IPR001762, which indicates the disintegrin domain (475-563), IPR034025, which indicates the catalytic domain 17 (223-477) ADAM10/ADAM17, IPR001590 peptidase M12B, and ADAM/reprolysin, and IPR002870, which indicates peptidase M12B propeptide (48-167; Figure 1).

**Figure 2.**
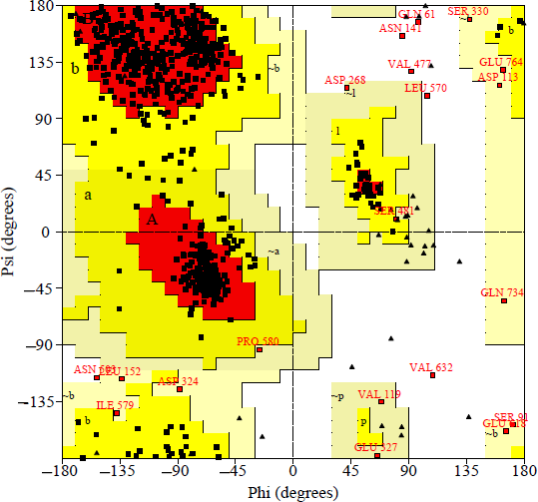
Ramachandran plot of the native model generated by PROCHECK. PROCHECK: Programs to Check the Stereochemical Quality of Protein Structures.

Total number of residues is 824. In the native ADAM17 structure, 604 (82.9%) of amino acids were in the favorable region, 119 (16.3%) in the allowed region, and 6 (0.8%) in the disallowed region. However, in the mutant structure (eg, Q658H), the percentage of the favorable region decreased, and the disallowed region increased, which can be explained by the fact that the mutation impacts the protein and its modeling. All Ramachandran plot results by PROCHECK are provided in Multimedia Appendices 2-6 (Figure 2 and Table 7).

**Table 7.** Percentage of different regions for each mutation using PROCHECK^a^.

Mutation	Favored region, n (%)	Allowed region, n (%)	Disallowed region, n (%)
Native	604 (82.9)	119 (16.3)	6 (0.8)
Q658H	585 (80)	129 (17.6)	17 (2.3)
D657G	608 (83.3)	112 (15.3)	10 (1.4)
D654N	600 (82.1)	121 (16.6)	10 (1.4)
F652L	609 (83.3)	108 (14.8)	14 (1.9)

a PROCHECK: Programs to Check the Stereochemical Quality of Protein Structures.

### Modeling of ADAM17 Using SWISS-MODEL and Sparks-X

In this study, we used the SWISS-MODEL server to construct the 3D structure of the native and 7 mutants of the ADAM17 protein. We used 2dw0.1. A (crystal structure of vesicle-associated membrane protein-associated protein 2 from Crotalus Atrox venom [Form 2‐1 crystal]) as a template with a sequence identity equal to 35.21% and resolution of 2.15 A°. In the second study, the Sparks-X server was used to generate the 3D structure of both native and the 4 mutants of ADAM17 molecules.

### Visualization of ADAM17 Mutations Using the PyMol Program

**Figure 3.**
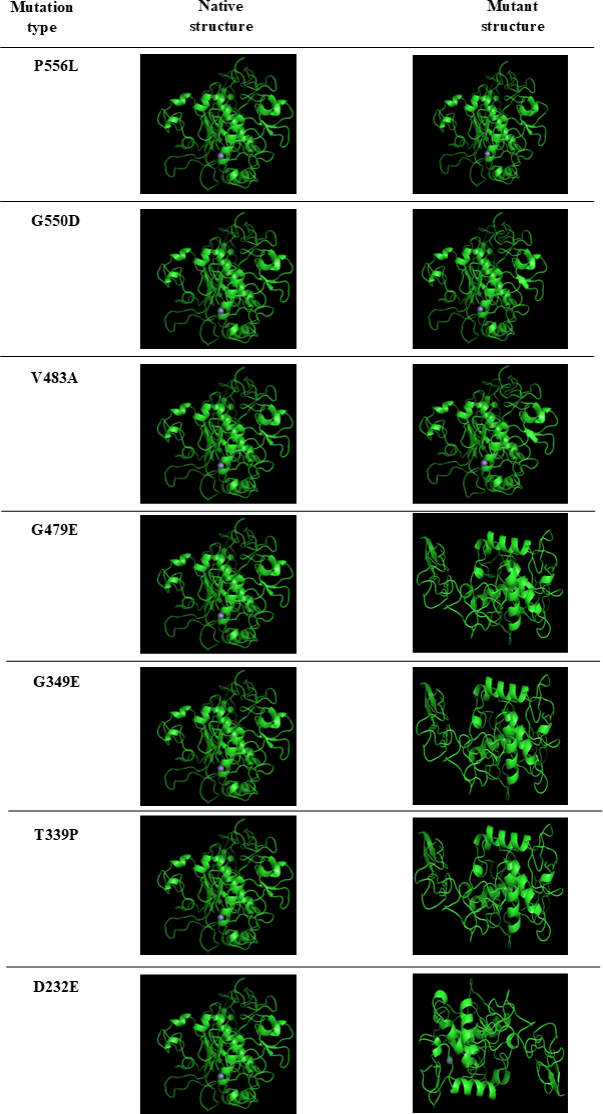
Visualization of ADAM17 native and mutations using the PyMol program. ADAM: A disintegrin and metalloprotease.

The 3D structures of the ADAM17 native and mutant models were visualized using PyMol. Structural similarities and differences between the ADAM17 native and its mutants are shown in Figures3  4.

**Figure 4.**
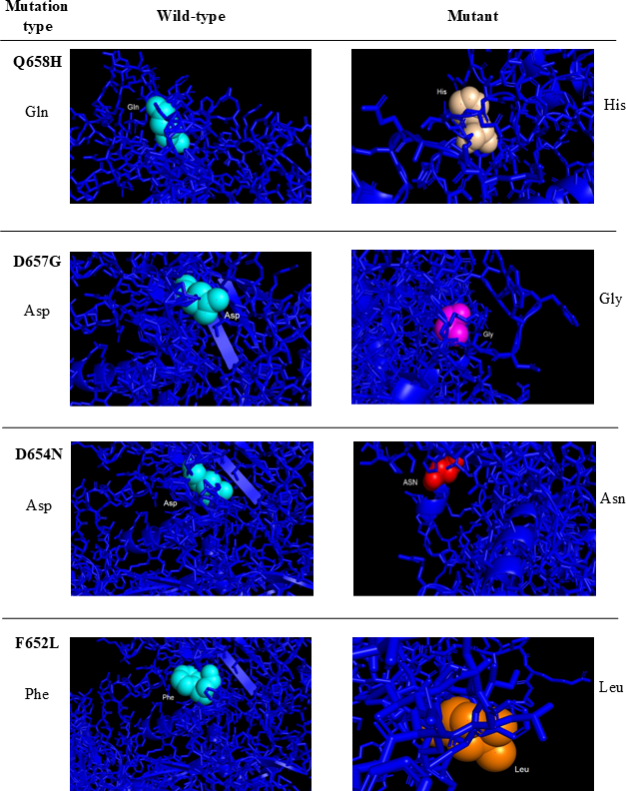
Visualization of ADAM17 native and mutations related to SARS-CoV-2 using the PyMol program. ADAM: A disintegrin and metalloprotease.

### Prediction of Structural Effects of Mutations in ADAM17 Using the HOPE Server

#### rs1394373815

The sizes are different between the amino acids of wild-type and mutant. The mutant residue is larger than the wild-type residue, and this might lead to bumps. Prolines are known to have a very rigid structure, sometimes forcing the backbone in a specific conformation. Possibly, the mutation changes a proline with such a function into another residue, thereby disturbing the local structure. The residue is found on the surface of the protein.

#### rs542316178

The charge of the mutant amino acid differs from that of the wild-type. The mutation introduces a charge, and this can cause repulsion of ligands or other residues with the same charge. The sizes are different between the amino acids of wild-type and mutant. The mutant residue is larger, and this might lead to bumps. The torsion angles for this residue are unusual. Only glycine is flexible enough to make these torsion angles, and mutation into another residue will force the local backbone into an incorrect conformation and will disturb the local structure.

#### rs777478676

The sizes are different between the amino acids of wild-type and mutant. The mutant residue is smaller, and this might lead to loss of interactions. The mutant residue is situated close to a position that is highly conserved. The mutation introduces an amino acid with different properties, which can disturb this domain and abolish its function.

#### rs951262662

The mutant amino acid carries a charge that differs from the wild-type counterpart. Because the mutation adds a charge, it can repel ligands or residues with similar charges. The sizes of the amino acids in the mutant and wild-type also differ, with the mutant residue being bulkier, which may result in steric hindrance. The torsion angles for this residue are unusual; glycine is the only amino acid flexible enough to adopt these angles. Mutation to a different residue will force the local backbone into an improper conformation and disturb the surrounding structure.

#### rs1192348585

The charge of the mutant amino acid contrasts with that of the wild-type. This mutation introduces a charge that may cause repulsive interactions with ligands or other residues carrying the same charge. Size differences between the mutant and wild-type amino acids are notable, as the mutant residue is larger and could cause clashes. The torsion angles for this residue are uncommon; glycine alone has the necessary flexibility to maintain such angles. Mutating to any other residue will impose strain on the local backbone, leading to an incorrect conformation and disruption of the local structural environment.

#### rs1157021454

The wild-type and mutant residues have different levels of hydrophobicity. At this location, the mutation adds a more hydrophobic residue. This may cause hydrogen bonds to break or disturb the proper. The wild-type residue and the mutant residue share certain properties. This mutation might occur in some rare cases, but it is more likely that the mutation is damaging to the protein.

#### rs768704961

The sizes are different between the amino acids of wild-type and mutant. The mutant residue is larger, and this might lead to bumps. The mutation is located within a domain and annotated in UniProt as peptidase M12B. Only this residue type was found at this position. Mutation of a 100% conserved residue is usually damaging for the protein.

## Discussion

### Principal Findings

In this study, different tools were used to identify the most deleterious nsSNPs of the ADAM17 protein, namely, SIFT, PolyPhen, PROVEAN, SNAP2, Mutation Assessor, I-Mutant, MUpro, and ConSurf; these tools were selected according to the following steps: pathogenicity study, stability, and conservation study. Parameters like accuracy, sensitivity, and specificity were chosen to assess their predictive abilities. Without these parameters, it will not be possible to completely evaluate the accuracy of a test.

In this bioinformatic study, we identified 7 nsSNPs (P556L, G550D, V483A, G479E, G349E, T339P, and D232E) from the entire residues of ADAM17. These nsSNPs were predicted by 5 tools: SIFT score of all these mutations ≈0 and classed as deleterious effect on the protein, PolyPhen score≈1 and classed in the probably damaging class, PROVEAN score of all of these mutations is negative (<−3.4) and was predicted deleterious, Mutation Assessor score of all of these mutations is positive (>3.7) and predicted to have a high functional impact on the protein, and SNAP2 score results were positive (>42) and classed to have a functional effect on the protein. In addition, we also evaluated protein stability using I-Mutant and MUpro. I-Mutant predicted that all 7 mutations would decrease the protein stability. MUpro results agreed for most mutations, except that the P556L mutation was predicted to increase the stability. Maximum conservation score by ConSurf means that all mutations were predicted to have functional effects, except the G349E mutation, which was predicted to have a structural effect on ADAM17.

These mutations (P556L, G550D, V483A, G479E, G349E, T339P, and D232E; rs1394373815, rs542316178, rs777478676, rs951262662, rs1192348585, rs1157021454, and rs768704961) are novel for their impact on ADAM17 structure, function, and stability.

The second part of this study focuses on nsSNPs that may be directly related to SARS-CoV-2 due to their positions within the ADAM17 protein. We analyzed 4 nsSNPs of interest (Q658H, D657G, D654N, and F652L), which were found to have the highest conservation scores and were predicted to be deleterious and reducing the stability of ADAM17. We hypothesized that these residues (Q658, D657, D654, and F652) are actively involved in the cleavage of ACE2 by ADAM17, and a mutation at any of these positions could disrupt the entire cleavage process. To support this hypothesis, we used a series of tools to assess the pathogenicity of these mutants.

### Comparison to Prior Studies

More than 80 distinct substrates have been discovered to be processed by ADAM17, also referred to as TNFα-converting enzyme, since its discovery. ADAM17, like most other ADAM relations, is understood to process single-spanning membrane proteins like growth factors, cytokines, receptors, chemokines, and regulators of neurological processes and diseases, and ADAM17 processes more than 80 substrates, and lots of them are linked to inflammatory and cancerous diseases. More recently, molecules important to tumor immunosurveillance have been found to be substrates for ADAM17, and research on the shedding events that this enzyme orchestrates has produced new theories of resistance to common cancer treatments. While ADAM17 features a wide range of substrate profiles, it typically only becomes active in response to triggers that cause disease states, making it a good target for a treatment approach.

The study by Pavlenko et al [49] has demonstrated that there are important ADAM17 residues, namely, R177C, D616N, D657A, and R725H, that play important roles in different cancer types. The R177C mutation affects the prodomain of ADAM17 and causes cecum and central nervous system cancer, the D616N affects the membrane-proximal domain and causes cancer in colon and uterus, the D657A residue affects the membrane-proximal domain and causes colon cancer, and R725H residue affects the cytoplasmic domain and causes colon cancer [49].

Mutations in the ADAM17 gene have been associated with neonatal inflammatory skin and bowel disease, a condition characterized by inflammatory features with neonatal onset, affecting the skin, hair, and gastrointestinal tract. The skin lesions involve perioral and perianal erythema, psoriasiform erythroderma, with flares of erythema, scaling, and widespread pustules. Gastrointestinal symptoms include malabsorptive diarrhea that is exacerbated by intercurrent gastrointestinal infections. The hair is brief or broken; therefore, the eyelashes and eyebrows are wiry and disorganized. The results of this study may be applicable for the analysis of novel missense variants of the ADAM17 gene.

Several studies have demonstrated that residues located between positions 652 and 659 catalyze the shedding of the ACE2 ectodomain by ADAM17 [50,51]. Recent advances in deep learning, such as self-supervised learning, provide promising avenues for enhancing the predictive capabilities of bioinformatics tools like the ones implemented in this work. In addition, application of federated learning with its privacy-preserving analytics approach applied to Internet of Things in smart health care could increase the scope for computational approaches such as that done for the ADAM17 variant [52,53].

### Limitations

Our study has several limitations. First, it is based entirely on computational analysis using predictive tools and servers, which may have many varying confidence levels and potential false positive rates that we did not fully address. In addition, the structural analysis using PyMol revealed visual differences between native and mutant proteins, but their functional implications of these structural changes are not thoroughly explored or explained. Second, the 7 deleterious variants and the 4 variants that have a relation with SARS-CoV-2 infection should be confirmed with future laboratory experiments and clinical wet laboratory approaches to figure out the mechanism of these mutations.

### Conclusions

In this in silico study of the high-risk missense variants of ADAM17, we identified 7 nsSNPs (P556L, G550D, V483A, G479E, G349E, T339P, and D232E) as the most deleterious mutations in the ADAM17 gene. All 7 mutations were predicted to have damaging effects on the structure, function, and stability of the ADAM17 protein. This study represents the first in silico analysis that evaluates the effect of these missense variants on the function and structure of ADAM17, and these results still require validation with in vitro experiments.

To support this study, in vitro experiments should be conducted to confirm the in silico results. For future research, our results confirm the impact of the 4 named mutations (Q658H, D657G, D654N, and F652L) on the pathology related to SARS-CoV-2, which strongly reinforces the role of ADAM17 in the ectodomain shedding process of ACE2.

Our findings form a basis for understanding the potential implications of ADAM17 variants on disease, which may lead to earlier diagnosis, assessment of risk for progression of related diseases, and may help inform future therapeutic targeting.

## Supplementary material

10.2196/72133 Multimedia Appendix 1 ConSurf results.

10.2196/72133 Multimedia Appendix 2 Ramachandran for ADAM17 wild. ADAM: A disintegrin and metalloprotease.

10.2196/72133 Multimedia Appendix 3 Ramachandran ADAM17 mutation D654N. ADAM: A disintegrin and metalloprotease.

10.2196/72133 Multimedia Appendix 4 Ramachandran ADAM17 mutation D657G. ADAM: A disintegrin and metalloprotease.

10.2196/72133 Multimedia Appendix 5 Ramachandran ADAM17 mutation F652L. ADAM: A disintegrin and metalloprotease.

10.2196/72133 Multimedia Appendix 6 Ramachandran ADAM17 mutation Q658H. ADAM: A disintegrin and metalloprotease.

## Abbreviations

ACE2: angiotensin-converting enzyme 2
ADAM: A disintegrin and metalloprotease domain
HOPE: Have (y)Our Protein Explained
nsSNP: nonsynonymous single-nucleotide polymorphism
PANTHER: Protein Analysis Through Evolutionary Relationships
PhD-SNP: Predictor of Human Deleterious Single Nucleotide Polymorphisms
PROCHECK: Programs to Check the Stereochemical Quality of Protein Structures
PROVEAN: Protein Variation Effect Analyzer
SIFT: Sorting Intolerant From Tolerant
SNAP2: Screening for Non-Acceptable Polymorphisms 2
SNP: single-nucleotide polymorphism
SNP&GO: Single Nucleotide Polymorphisms and Gene Ontology
SVM: support vector machine
TMPRSS2: transmembrane protease, serine 2
TNF-α: tumor necrosis factor alpha
