# Supplementary material for: In Silico Analysis and Validation of A Disintegrin and Metalloprotease (ADAM) 17 Gene Missense Variants: Structural Bioinformatics Study
Source: JMIR Bioinform Biotechnol. 2025 Aug 25;6:e72133. doi: 10.2196/72133 (PMC12377791; doi:10.2196/72133)
Supplement: Multimedia Appendix 1 [file bioinform-v6-e72133-s001.pdf]

```
1
M R Q S L L F L T S
e e e b b b b b b b
f

11
V V P F V L A P R P
b b b b b b b b e e

21
P D D P G F G P H Q
e e e e e e e e e e

31
R L E K L D S L L S
e b e e b e e b b e
                s

41
D Y D I L S L S N I
e b e b b e b e e b

51
Q Q H S V R K R D L
e e e e b e e e e e
    f       f f f

61
Q T S T H V E T L L
e e e e e e e e e b
            f

71
T F S A L K R H F K
e b e b b e e e b e
  s         f   s

81
L Y L T S S T E R F
b b b e e e e e e b
    s

91
S Q N F K V V V V D
e e e b e b b b b e
      s

101
G K N E S E Y T V K
e e e e e e e e b e

111
W Q D F F T G H V V
e e e b b e b e b e
            s f

121
G E P D S R V L A H
e e e e e e b e b b
f f     f

131
I R D D D V I I R I
b e e e e b e b e b
            f     s

141
N T D G A E Y N I E
b b e e e e e e b e
          f   f   f

151
P L W R F V N D T K
e b b e b b e e e e
f   s f

161
D K R M L V Y K S E
e e e b b b b e e e
            s     f

171
D I K N V S R L Q S
e b e e b e e e e e
f

181
P K V C G Y L K V D
e e b b e e e e e e
      s   f

191
N E E L L P K G L V
e e e e e e e e e e

201
D R E P P E E L V H
e e e e e e e e e e

211
R V K R R A D P D P
e b e e e e e e e e
f       f

221
M K N T C K L L V V
e e e e b e b b b b
    f f s     s s s

231
A D H R F Y R Y M G
b e b e b b e e b e
s f   f s       s f

241
R G E E S T T T N Y
e e e e e b b b e b
      f f   s   f s

251
L I E L I D R V D D
b b e b b e e b b e
s s f s   f f s   f

261
I Y R N T S W D N A
b b e e e e e e e e
s s f   f   f

271
G F K G Y G I Q I E
e b e e e b b e b e
      f f   s f s

281
Q I R I L K S P Q E
e b b b e e e e e e
f s     f       f

291
V K P G E K H Y N M
e e e e e e e b e b
            f   f

301
A K S Y P N E E K D
e e e e e e e e e e
    f   f

311
A W D V K M L L E Q
e e e b e e b b e e
  f   s f   s s f f

321
F S F D I A E E A S
b b e e b b e e b e
s s

331
K V C L A H L F T Y
e b b b b b b b e b
    s s s s s s f

341
Q D F D M G T L G L
e e b e e b b b b b
f f s       s s s s

351
A Y V G S P R A N S
b b b b e e e e e e
s s s   f   f

361
H G G V C P K A Y Y
e e b b b e e e e e
  f s   s

371
S P V G K K N I Y L
e e e e e e e b b b
                  s

381
N S G L T S T K N Y
b e e e e e e e e e
s f f f f f f   f f

391
G K T I L T K E A D
b e e b b e e e b e
  f f s s f f f s f

401
L V T T H E L G H N
b b b b e e b b b e
s s s s f f s s s f

411
F G A E H D P D G L
b e e e e e e e e b
s f f f f f f f

421
A E C A P N E D Q G
e e b e e e e e e e
    s f f   f     f

431
G K Y V M Y P I A V
e e b b b b b b b b
f f     s s s s s s

441
S G D H E N N K M F
b b e e e e e e e b
s s f     f f f   s

451
S N C S K Q S I Y K
e e b b e e b b e e
f   s s

461
T I E S K A Q E C F
e b e e e b e e b b
f s     f       s s

471
Q E R S N K V C G N
e e e e e e b b e e
    f         s f f

481
S R V D E G E E C D
b e e e e e e e b e
  f f f     f   s f

491
P G I M Y L N N D T
e e b b e e e e e e
f f             f

501
C C N S D C T L K E
b b e e e b e b e e
s s       s

511
G V Q C S D R N S P
e e e b e e e e e e
      s f f f f f

521
C C K N C Q F E T A
e b e e b e e e e e
f s     s

531
Q K K C Q E A I N A
e e e b e e e e e e
      s f

541
T C K G V S Y C T G
e b e e e b e b e e
f s f         s   f

551
N S S E C P P P G N
e e e e e e e e e e
        f f   f

561
A E D D T V C L D L
e e e e e e b e e e
            s   f

571
G K C K D G K C I P
e e b e e e e b e e
f   s     f   s   f

581
F C E R E Q Q L E S
e b e e e e e b e e
f s f             f

591
C A C N E T D N S C
b b b e e e e e e b
s   s f         f s
```

```
601                  611                  621                  631                  641
K V C C R D L S G R  C V P Y V D A E Q K  N L F L R K G K P C  T V G F C D M N G K  C E K R V Q D V I E
e e b b e e e e e e  b e e e e e e e e e  b e b b e e e e e e  e e b e e e e e e e  e e e e b e e b b e
  f s s              s   f                          f f f f f  f f s f f       f    f f f f   f f     f

651                  661                  671                  681                  691
R F W D F I D Q L S  I N T F G K F L A D  N I V G S V L V F S  L I F W I P F S I L  V H C V D K K L D K
e b b e b b e e b e  b e e b b e b b e e  e b b b b b b b b b  b b b b b b b b b b  b b b b e e e e e e
f   s     s f f   f    f             f    f   s     s     s        s   s   s              f

701                  711                  721                  731                  741
Q Y E S L S L F H P  S N V E M L S S M D  S A S V R I I K P F  P A P Q T P G R L Q  P A P V I P S A P A
e e e e e e e e e e  e e e e b b e e b e  e b b b e b b e e e  e e e e e e e e e e  e e e e e e e e e e
                     f

751                  761                  771                  781                  791
A P K L D H Q R M D  T I Q E D P S T D S  H M D E D G F E K D  P F P N S S T A A K  S F E D L T D H P V
e e e e e e e e b e  e b e e e e e e e e  e b e e e e e e e e  e e e e e e e e e e  b b e e b e e e e e
                s    f s   f f   f     f                         f                  s   f f s f

801                  811                  821
T R S E K A A S F K  L Q R Q N R V D S K  E T E C
e e e e e e e b b e  b e e e e e e e e e  e e e e
                         f f           f  f f   f
```

The conservation scale:

?	1	2	3	4	5	6	7	8	9

Variable Average Conserved

e - An exposed residue according to the neural-network algorithm.
b - A buried residue according to the neural-network algorithm.
f - A predicted functional residue (highly conserved and exposed).
s - A predicted structural residue (highly conserved and buried).
X - Insufficient data - the calculation for this site was performed on less than 10% of the sequences.
