# Supplementary material for: In Silico Analysis and Validation of A Disintegrin and Metalloprotease (ADAM) 17 Gene Missense Variants: Structural Bioinformatics Study
Source: JMIR Bioinform Biotechnol. 2025 Aug 25;6:e72133. doi: 10.2196/72133 (PMC12377791; doi:10.2196/72133)
Supplement: Multimedia Appendix 2 [file bioinform-v6-e72133-s002.pdf]

PROCHECK

Ramachandran Plot

saves

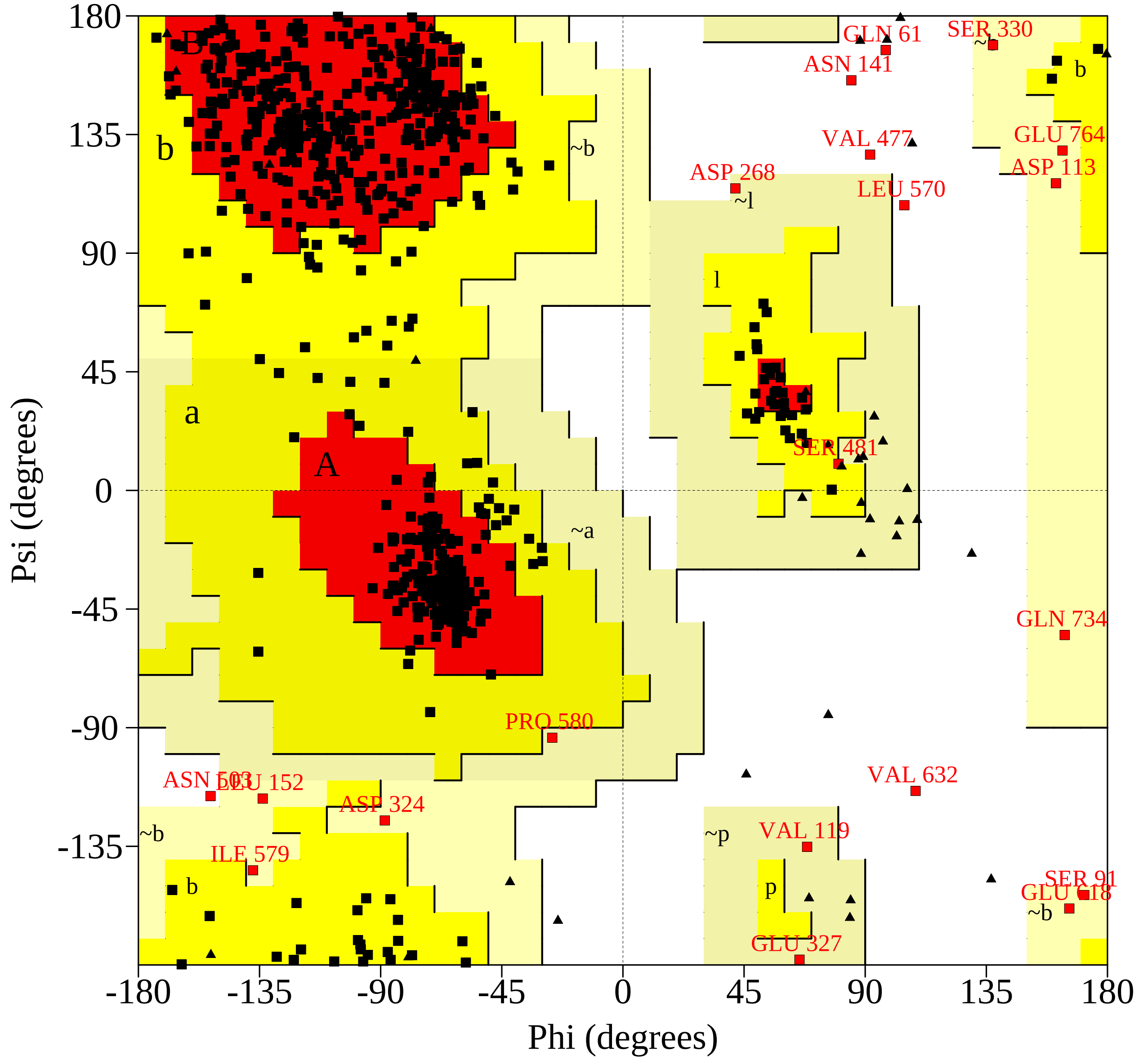

Plot statistics

Residues in most favoured regions [A,B,L]	604	82.9%
Residues in additional allowed regions [a,b,l,p]	106	14.5%
Residues in generously allowed regions [~a,~b,~l,~p]	13	1.8%
Residues in disallowed regions	6	0.8%
	----	------
Number of non-glycine and non-proline residues	729	100.0%
Number of end-residues (excl. Gly and Pro)	4	
Number of glycine residues (shown as triangles)	45	
Number of proline residues	46	

Total number of residues	824	

Based on an analysis of 118 structures of resolution of at least 2.0 Angstroms and R-factor no greater than 20%, a good quality model would be expected to have over 90% in the most favoured regions.

saves_01.ps
